# Supplementary material for: Quality of care in a differentiated HIV service delivery intervention in Tanzania: A mixed-methods study
Source: PLoS One. 2022 Mar 15;17(3):e0265307. doi: 10.1371/journal.pone.0265307 (PMC8923447; doi:10.1371/journal.pone.0265307)
Supplement: S1 Appendix — (PDF) [file pone.0265307.s001.pdf]

Participant unique ID number　　　　　　　　　　Date

□□-□□□□　　　　　　　　　　□□ / □□ /□□□□

Site number-Patient number　　　　　　**DD　MM　YYYY**

Appendix I (Patient)　　　　　　**Research QUESTIONNAIRE**

Yafuatayo ni mambo ambayo wagonjwa walio na ugonjwa kama wako huona ni muhimu. **Tafadhali chagua jawabu mwafaka linalokuhusu <u>kwa siku saba zilizopita</u>.**

Section III: Quality of care

Part A: Outcomes of care for HIV care delivery in Clinic or Club (Adapted from QUOTE-HIV)				
1.	Which Health Care provider (HCP) attended to you today	❑ Doctor ❑ Clinical Officer ❑ Nurse<br>❑ Pharmacist/Dispenser ❑ Lab scientist<br>❑ HBC/Lay counsellor ❑ Records officer<br>Others ❑ ❑		
2.	Which HCP did you spend most of the time with			
	**Provide responses to the following statements based on your experience with the following HCP**	**Doctor/Nurse/ Clinical officer**	**Pharmacist/Dispenser**	**HBC/ Lay Counsellor**
3.	**i. Content of care**<br>My HCP explains the advantages and disadvantages of taking my ARV regularly.	❑ Never<br>❑Occasionally<br>❑ Mostly<br>❑ Always	❑<br>❑<br>❑<br>❑	❑<br>❑<br>❑<br>❑
4.	My HCP ensures I get my ARV supply regularly and conveniently	❑ Never<br>❑Occasionally<br>❑ Mostly<br>❑ Always	❑<br>❑<br>❑<br>❑	❑<br>❑<br>❑<br>❑
5. .	My HCP notifies me about the results of my laboratory Tests when necessary	❑ Never<br>❑Occasionally<br>❑ Mostly<br>❑ Always	❑<br>❑<br>❑<br>❑	❑<br>❑<br>❑<br>❑
6.	My HCP is able to answer any questions I have about HIV	❑ Never<br>❑Occasionally<br>❑ Mostly<br>❑ Always	❑<br>❑<br>❑<br>❑	❑<br>❑<br>❑<br>❑
7.	My HCP keeps on informing me about ways to prevent spreading the virus and re-infection	❑ Never<br>❑Occasionally<br>❑ Mostly<br>❑ Always	❑<br>❑<br>❑<br>❑	❑<br>❑<br>❑<br>❑
8.	My HCP is willing to talk with me about anything that makes me sad	❑ Never<br>❑Occasionally<br>❑ Mostly<br>❑ Always	❑<br>❑<br>❑<br>❑	❑<br>❑<br>❑<br>❑
9.	**ii. Information**<br>My HCP explains in language that I understand what the possible side effects of my ARV are when necessary.	❑ Never<br>❑Occasionally<br>❑ Mostly<br>❑ Always	❑<br>❑<br>❑<br>❑	❑<br>❑<br>❑<br>❑
10.	**iii. Relationship with the HCW**<br>My HCP listens to me and takes me seriously.	❑ Never<br>❑Occasionally<br>❑ Mostly<br>❑ Always	❑<br>❑<br>❑<br>❑	❑<br>❑<br>❑<br>❑
11.	My HCP knows how to break bad news about my health carefully (e.g. High viral load, low CD4, Weight loss etc.)	❑ Never<br>❑Occasionally<br>❑ Mostly<br>❑ Always	❑<br>❑<br>❑<br>❑	❑<br>❑<br>❑<br>❑
12.	My HCP takes enough time to talk to me.	❑ Never<br>❑Occasionally<br>❑ Mostly<br>❑ Always	❑<br>❑<br>❑<br>❑	❑<br>❑<br>❑<br>❑
13.	My HCP is aware of the situation with me	❑ Never	❑	❑

Initial & Date (Research Staff): ___________________ Initial & Date (Data Staff): ________________________

	at home and at work / school.	❑ Occasionally<br>❑ Mostly<br>❑ Always	❑<br>❑<br>❑	❑<br>❑<br>❑
14.	My HCP is friendly, I find it easy to share anything with my HCP	❑ Never<br>❑ Occasionally<br>❑ Mostly<br>❑ Always	❑<br>❑<br>❑<br>❑	❑<br>❑<br>❑<br>❑
15.	I feel afraid to tell my HCP somethings happening to me	❑ Never<br>❑ Occasionally<br>❑ Mostly<br>❑ Always	❑<br>❑<br>❑<br>❑	❑<br>❑<br>❑<br>❑
16.	**iv. Organization of care**<br>My HCP treats me well	❑ Never<br>❑ Occasionally<br>❑ Mostly<br>❑ Always	❑<br>❑<br>❑<br>❑	❑<br>❑<br>❑<br>❑
17.	My HCP works well with other health workers (staff)	❑ Never<br>❑ Occasionally<br>❑ Mostly<br>❑ Always	❑<br>❑<br>❑<br>❑	❑<br>❑<br>❑<br>❑
18.	My HCP is easily accessible by telephone	❑ Never<br>❑ Occasionally<br>❑ Mostly<br>❑ Always	❑<br>❑<br>❑<br>❑	❑<br>❑<br>❑<br>❑
19.	My HCP ensure that I do not stay for longer than necessary during CTC clinic visit /ARV club meeting	❑ Never<br>❑ Occasionally<br>❑ Mostly<br>❑ Always	❑<br>❑<br>❑<br>❑	❑<br>❑<br>❑<br>❑
20.	My HCP arranges/reminds me of appointments for CTC clinic visit / ARV club meeting adequately ***(scheduled properly ahead of time so I can make arrangement to come)***	❑ Never<br>❑ Occasionally<br>❑ Mostly<br>❑ Always	❑<br>❑<br>❑<br>❑	❑<br>❑<br>❑<br>❑
21.	My HCP makes sure that if necessary, I get appropriate referral when I need it	❑ Never<br>❑ Occasionally<br>❑ Mostly<br>❑ Always	❑<br>❑<br>❑<br>❑	❑<br>❑<br>❑<br>❑
22.	With my HCP I can talk undisturbed during consultation	❑ Never<br>❑ Occasionally<br>❑ Mostly<br>❑ Always	❑<br>❑<br>❑<br>❑	❑<br>❑<br>❑<br>❑
23.	My HCP makes effort to reach me when I don't show up for my appointment<br>*Mark **X** here ❑ if patient has never missed appointment	❑ Never<br>❑ Occasionally<br>❑ Mostly<br>❑ Always	❑<br>❑<br>❑<br>❑	❑<br>❑<br>❑<br>❑
24.	**v. Patient right and privacy**<br>My HCP regards my opinion in my care	❑ Never<br>❑ Occasionally<br>❑ Mostly<br>❑ Always	❑<br>❑<br>❑<br>❑	❑<br>❑<br>❑<br>❑
25.	I feel free to verify any advice I receive from another health worker with my HCP	❑ Never<br>❑ Occasionally<br>❑ Mostly<br>❑ Always	❑<br>❑<br>❑<br>❑	❑<br>❑<br>❑<br>❑

26.	My HCP tells me anything I want to know about my medical file or refers me to the Doctor	❑ Never<br>❑ Occasionally<br>❑ Mostly<br>❑ Always	❑<br>❑<br>❑<br>❑	❑<br>❑<br>❑<br>❑
27.	My HCP guarantees privacy about my HIV status.	❑ Never<br>❑ Occasionally<br>❑ Mostly<br>❑ Always	❑<br>❑<br>❑<br>❑	❑<br>❑<br>❑<br>❑
28.	I'm afraid to tell my HCP about something happening in my life because I feel she will tell others about it	❑ Never<br>❑ Occasionally<br>❑ Mostly<br>❑ Always	❑<br>❑<br>❑<br>❑	❑<br>❑<br>❑<br>❑
29.	My HCP has arranged the meeting space in such a way that no one can hear when I am talking with her in confidence.	❑ Never<br>❑ Occasionally<br>❑ Mostly<br>❑ Always	❑<br>❑<br>❑<br>❑	❑<br>❑<br>❑<br>❑
**Part B: Outcomes of care (IOM dimensions of quality)**				
**Capture patients experience in the last 3 CTC clinic visits/ARV club meetings**				
		**Visit 1**	**Visit 2**	**Visit 3**
30.	**Access to services:**<br>I find it easy to attend CTC clinic/ARV Club meeting	❑ Yes<br>❑ No	❑ Yes<br>❑ No	❑ Yes<br>❑ No
31.	**Acceptability /patient-centeredness:**<br>I like the way I am treated by the staff during my visit	❑ Yes<br>❑ No	❑ Yes<br>❑ No	❑ Yes<br>❑ No
32.	I am satisfied with the way my HCP organizes the CTC Clinic/ARV Club meeting	❑ Yes<br>❑ No	❑ Yes<br>❑ No	❑ Yes<br>❑ No
33.	**Effectiveness:**<br>My HCP convinces me to use other alternative treatments e.g. local herbs which I have to pay for	❑ Yes<br>❑ No	❑ Yes<br>❑ No	❑ Yes<br>❑ No
34.	**Efficient:**<br>We spend a lot of time in the CTC clinic/Club meetings	❑ Yes<br>❑ No	❑ Yes<br>❑ No	❑ Yes<br>❑ No
35.	I consider the time spent in the CTC clinic/ARV Club as a waste of time	❑ Yes<br>❑ No	❑ Yes<br>❑ No	❑ Yes<br>❑ No
36.	Average time spent during your last 3 visit/ meeting	1st	2nd	3rd
37.	**Equity:**<br>I feel I am not treated as well as other patients during CTC Clinic/ARV club meetings	❑ Yes<br>❑ No	❑ Yes<br>❑ No	❑ Yes<br>❑ No
38.	**Patient safety:**<br>I know the kind of ARV I am using	❑ Yes<br>❑ No	❑ Yes<br>❑ No	❑ Yes<br>❑ No
39.	If Yes to 194, which ARV? ***(e.g. TDF/FTC/EFV)***<br>*(Allow patient to describe ARV tablet and dosage)*			
40.	I receive the correct ARV every time I come	❑ Yes<br>❑ No	❑ Yes<br>❑ No	❑ Yes<br>❑ No
41.	I have been given the wrong ARV before	[ ] Never	[ ] Once	[ ] >1
42.	I have been given alternative ARV before	❑ Yes<br>❑ No		
43.	If Yes to 199, why? ***(e.g. drug stock out, drug reaction, pregnancy, TB etc.)***			
44.	I can discuss any concerns related to my treatment freely	❑ Yes<br>❑ No	❑ Yes<br>❑ No	❑ Yes<br>❑ No
45.	My HCP discusses any changes in my medication in a way that does not make me afraid	❑ Yes<br>❑ No	❑ Yes<br>❑ No	❑ Yes<br>❑ No

Appendix I (Patient) **Research QUESTIONNAIRE**

**Part C: Outcomes of care from Patient management records in CTC2: (last 3 Clinic visits/ ARV Club meetings)**		**Visit 1**	**Visit 2**	**Visit 3**
46.	Visit date documented	[ ]Yes[ ]No	[ ]Yes[ ]No	[ ]Yes[ ]No
47.	Type of visit documented<br>*(Scheduled visit – **SV**; Unscheduled Visit – **UV; Drug** Pick-up – **DPU**; Refill visit for stable client – **RV**)*	[ ]SV<br>[ ]UV<br>[ ]DPU<br>[ ]RV	[ ]SV<br>[ ]UV<br>[ ]DPU<br>[ ]RV	[ ]SV<br>[ ]UV<br>[ ]DPU<br>[ ]RV
48.	Weight documented *(write weight readings)*			
49.	Any unusual weight readings?	[ ]Yes[ ]No	[ ]Yes[ ]No	[ ]Yes[ ]No
50.	OIs documented	[ ]Yes[ ]No	[ ]Yes[ ]No	[ ]Yes[ ]No
51.	Any OIs? *(write OI documented)*			
52.	Functional status documented	[ ]Yes[ ]No	[ ]Yes[ ]No	[ ]Yes[ ]No
53.	Pregnancy documented (*for female clients*)	[ ]Yes[ ]No	[ ]Yes[ ]No	[ ]Yes[ ]No
54.	Pregnant? NA – Not Applicable	[ ]Yes[ ]No [ ]NA	[ ]Yes[ ]No [ ]NA	[ ]Yes[ ]No [ ]NA
55.	TB screening documented	[ ]Yes[ ]No	[ ]Yes[ ]No	[ ]Yes[ ]No
56.	TB suspected?	[ ]Yes[ ]No	[ ]Yes[ ]No	[ ]Yes[ ]No
57.	IPT dispensed and documented	[ ]Yes[ ]No [ ]NA	[ ]Yes[ ]No [ ]NA	[ ]Yes[ ]No [ ]NA
58.	ARV regimen dispensed documented	[ ]Yes[ ]No	[ ]Yes[ ]No	[ ]Yes[ ]No
59.	Adherence assessed and documented	[ ]Yes[ ]No	[ ]Yes[ ]No	[ ]Yes[ ]No
60.	Cotrimoxazole dispensed and documented	[ ]Yes[ ]No	[ ]Yes[ ]No	[ ]Yes[ ]No
61.	Nutritional status assessed and documented	[ ]Yes[ ]No	[ ]Yes[ ]No	[ ]Yes[ ]No
62.	Referrals documented *(**Referred to column is filled**)*	[ ]Yes[ ]No	[ ]Yes[ ]No	[ ]Yes[ ]No
63.	Where referred to<br>***PMTCT** (Pregnancy); **Support group/Club** (Peer support); **CTC clinic** (OI/Ill health); **Nutritional support** (Rapid weight loss); **TB** (Tuberculosis suspect)*	[ ] PMTCT<br>[ ] ARV club<br>[ ] CTC clinic<br>[ ] Nutritional<br>[ ] TB	[ ] PMTCT<br>[ ] ARV club<br>[ ] CTC clinic<br>[ ] Nutritional<br>[ ] TB	[ ] PMTCT<br>[ ] ARV club<br>[ ] CTC clinic<br>[ ] Nutritional<br>[ ] TB
64.	Follow-up status documented	[ ]Yes[ ]No	[ ]Yes[ ]No	[ ]Yes[ ]No
65.	Complete documentation for visit *(all required columns completed in register)*	[ ]Yes[ ]No	[ ]Yes[ ]No	[ ]Yes[ ]No
66.	CD4 count value at ART initiation *(write the value)*			
67.	CD4 count value in the last 6 months *(write the value)*			
68.	VL load in the last 6 months	[ ]Yes[ ]No	[ ]Yes[ ]No	[ ]Yes[ ]No
69.	VL result *(write the value)*			

Below are some general questions about the care you receive from your HCP.

70. Is the care you receive from your HCP for you:

- ☐ Satisfactory
- ☐ Not satisfactory
- ☐ Neither good or bad

71. You can indicate on the line below with a cross (X) how satisfied you are generally about the care you have received from your HCP in the past 6 months in connection with your HIV infection

- ☐ Satisfied
- ☐ Very satisfied
- ☐ Dissatisfied
- ☐ Very dissatisfied

Initial & Date (Research Staff): ____________________ Initial & Date (Data Staff): _________________________
