# Supplementary material for: Quality of care in a differentiated HIV service delivery intervention in Tanzania: A mixed-methods study
Source: PLoS One. 2022 Mar 15;17(3):e0265307. doi: 10.1371/journal.pone.0265307 (PMC8923447; doi:10.1371/journal.pone.0265307)
Supplement: S2 Appendix — (PDF) [file pone.0265307.s002.pdf]

Mradi wa UTT Shinyanga

Namba ya Utambulisho ya Mshiriki

□□-□□□□

Namba ya kituo-Namba ya Mgonjwa

Tarehe

□□ / □□ /□□□□

SS MM MMMM

Kiambatanisho I (Mgonjwa)

DODOSO LA UTAFITI

Mradi wa Upimaji na Matibabu (UTT) kwenye wilaya za Shinyanga na Simiyu, Tanzania

Yafuatayo ni mambo ambayo wagonjwa walio na ugonjwa kama wako huona ni muhimu. **Tafadhali chagua jawabu mwafaka linalokuhusu <u>kwa siku saba zilizopita</u>.**

<u>Sehemu ya III: Ubora wa huduma</u>

**Sehemu A: Matokeo ya Utoaji huduma za VVU ndani ya Kliniki au Klabu (Iliyotokana na QUOTE-VVU)**				
1.	Je, leo ulihudumiwa na aina gani ya mtoa huduma za afya?	❑ Daktari ❑ Daktari msaidizi ❑ Muuguzi<br>❑ Mfamasia/mtoa dawa ❑Mtaalam wa maabara<br>❑ Mhudumu wa afya majumbani/Mshauri nasaha asiye na mafunzo rasmi<br>❑ Mtunza kumbukumbu<br>Nyingine ❑ ❑		
2.	Je, ulitumia muda mwingi Zaidi na aina gani ya mtoa huduma za afya?			
	**Toa majibu kwenye kauli zifuatazo kulingana na uzoefu wako kwa kila mtoa huduma wa afya kati ya hawa walioorodheshwa kulia**	**Daktari/ Muuguzi/ Daktari msaidizi**	**Mfamasia/ Mtoa dawa**	**Mhudumu wa afya majumbani/ Mshauri nasaha asiye na mafunzo rasmi**
3.	**i. Maudhui ya huduma**<br>Mhudumu wangu wa afya huelezea faida na hasara za kutumia dawa zangu za kuzuia makali ya VVU mara kwa mara.	❑ Hajawahi<br>❑Mara chache<br>❑ Mara nyingi<br>❑ Daima	❑<br>❑<br>❑<br>❑	❑<br>❑<br>❑<br>❑
4.	Mhudumu wangu wa afya anahakikisha kwamba ninapata dawa zangu za kupunguza makali ya VVU mara kwa mara na kwa urahisi.	❑ Hajawahi<br>❑Mara chache<br>❑ Mara nyingi<br>❑ Daima	❑<br>❑<br>❑<br>❑	❑<br>❑<br>❑<br>❑
5.	Mhudumu wangu wa afya hunitaarifu kuhusu matokeo ya vipimo vyangu vya maabara kila kukiwa na umuhimu wa kufanya hivyo.	❑ Hapana kamwe<br>❑Mara chache<br>❑ Mara nyingi<br>❑ Daima	❑<br>❑<br>❑<br>❑	❑<br>❑<br>❑<br>❑
6.	Mhudumu wangu wa afya anaweza kujibu swali lolote nililonalo kuhusu VVU.	❑ Hapana kamwe<br>❑Mara chache<br>❑ Mara nyingi<br>❑ Daima	❑<br>❑<br>❑<br>❑	❑<br>❑<br>❑<br>❑
7.	Mhudumu wangu wa afya huendelea kunipa taarifa kuhusu njia za kuzuia kusambaza virusi na kuzuia maambukizi mapya.	❑ Hapana kamwe<br>❑Mara chache<br>❑ Mara nyingi<br>❑ Daima	❑<br>❑<br>❑<br>❑	❑<br>❑<br>❑<br>❑
8.	Mhudumu wangu wa afya yuko tayari kuzungumza na mimi kuhusu kitu chochote ambacho kinanihuzunisha.	❑ Hapana kamwe<br>❑Mara chache<br>❑ Mara nyingi<br>❑ Daima	❑<br>❑<br>❑<br>❑	❑<br>❑<br>❑<br>❑
9.	**ii. Taarifa**<br>Mhudumu wangu wa afya hunielezea kwa lugha ya kueleweka madhara yanayoweza kutokea kutokana na	❑ Hapana kamwe<br>❑Mara chache<br>❑ Mara nyingi	❑<br>❑<br>❑	❑<br>❑<br>❑

Initial & Date (Research Staff): ____________________ Initial & Date (Data Staff): __________________________

	kutumia dawa za kupunguza makali ya VVU pale inapobidi.	❑ Daima	❑	❑
10.	**iii. Uhusiano na mhudumu wa afya**<br>Mhudumu wangu wa afya hunisikiliza na kunichukulia kwa uzito.	❑ Hapanakamwe❑ Mara chache<br>❑ Mara nyingi<br>❑ Daima	❑<br>❑<br>❑<br>❑	❑<br>❑<br>❑<br>❑
11.	Mhudumu wangu wa afya anafahamu namna ya kunipa taarifa mbaya kuhusu afya yangu kwa umakini (m.f. wingi wa virusi, CD4 zikiwa chini, kupungua uzito n.k.)	❑ Hapana kamwe<br>❑Mara chache<br>❑ Mara nyingi<br>❑ Daima	❑<br>❑<br>❑<br>❑	❑<br>❑<br>❑<br>❑
12.	Mhudumu wangu wa afya anatumia muda wa kutosha kuzungumza na mimi.	❑ Hapana kamwe<br>❑Mara chache<br>❑ Mara nyingi<br>❑ Daima	❑<br>❑<br>❑<br>❑	❑<br>❑<br>❑<br>❑
13.	Mhudumu wangu wa afya anafahamu kuhusu hali niliyonayo nyumbani na kazini/shuleni.	❑ Hapana kamwe<br>❑Mara chache<br>❑ Mara nyingi<br>❑ Daima	❑<br>❑<br>❑<br>❑	❑<br>❑<br>❑<br>❑
14.	Mhudumu wangu wa afya ni rafiki, ninaona ni rahisi kumshirikisha chochote.	❑ Hapana kamwe<br>❑Mara chache<br>❑ Mara nyingi<br>❑ Daima	❑<br>❑<br>❑<br>❑	❑<br>❑<br>❑<br>❑
15.	Ninaogopa kumwambia mhudumu wangu wa afya kuhusu baadhi ya vitu ambavyo vinanitokea.	❑ Hapana kamwe<br>❑Mara chache<br>❑ Mara nyingi<br>❑ Daima	❑<br>❑<br>❑<br>❑	❑<br>❑<br>❑<br>❑
16.	**iv. Mpangilio wa huduma**<br>Mhudumu wangu wa afya hunihudumia vizuri.	❑ Hapana kamwe<br>❑Mara chache<br>❑ Mara nyingi<br>❑ Daima	❑<br>❑<br>❑<br>❑	❑<br>❑<br>❑<br>❑
17.	Mhudumu wangu wa afya anafanya kazi vizuri na watoa huduma wengine wa afya (watumishi)	❑ Hapana kamwe<br>❑Mara chache<br>❑ Mara nyingi<br>❑ Daima	❑<br>❑<br>❑<br>❑	❑<br>❑<br>❑<br>❑
18.	Mhudumu wangu wa afya anaweza kupatikana kwenye simu kwa urahisi.	❑ Hapana kamwe<br>❑Mara chache<br>❑ Mara nyingi<br>❑ Daima	❑<br>❑<br>❑<br>❑	❑<br>❑<br>❑<br>❑
19.	Mhudumu wangu wa afya anahakikisha kuwa situmii muda mrefu zaidi kuliko inavyotakiwa ninapotembelea kliniki ya huduma na tiba/kwenye	❑ Hapana kamwe<br>❑Mara chache<br>❑ Mara nyingi	❑<br>❑<br>❑	❑<br>❑<br>❑

	kikao cha klabu ya wanaotumia dawa za kupunguza makali ya VVU.	❑ Daima	❑	❑
20.	Mhudumu wangu wa afya hupanga/hunikumbusha tarehe za kutembelea kliniki ya huduma na tiba/kikao cha klabu ya wanaotumia dawa za kupunguza makali ya VVU kwa jinsi inavyofaa<br>***(tarehe hupangwa mapema vya kutosha ili kuniwezesha kufanya mipango ya kufika)***	❑ Hapana kamwe<br>❑Mara chache<br>❑ Mara nyingi<br>❑ Daima	❑<br>❑<br>❑<br>❑	❑<br>❑<br>❑<br>❑
21.	Mhudumu wangu wa afya anahakikisha kwamba kama ni muhimu, ninapata rufaa pale ninapoihitaji.	❑ Hapana kamwe<br>❑Mara chache<br>❑ Mara nyingi<br>❑ Daima	❑<br>❑<br>❑<br>❑	❑<br>❑<br>❑<br>❑
22.	Nikiwa na mhudumu wangu wa afya, ninaweza kuzungumza bila kusumbuliwa kipindi ambacho ananipa huduma.	❑ Hapana kamwe<br>❑Mara chache<br>❑ Mara nyingi<br>❑ Daima	❑<br>❑<br>❑<br>❑	❑<br>❑<br>❑<br>❑
23.	Mhudumu wangu wa afya hujitahidi kunitafuta pale ambapo sihudhurii kwenye tarehe niliyopangiwa kufika<br>*Weka alama ya **X** hapa ❑ kama mgonjwa hajawahi kukosa kufika kwenye tarehe aliyopangiwa	❑ Hapana kamwe<br>❑Mara chache<br>❑ Mara nyingi<br>❑ Daima	❑<br>❑<br>❑<br>❑	❑<br>❑<br>❑<br>❑
24.	**v. Haki za mgonjwa na faragha**<br>Mhudumu wangu wa afya husikiliza maoni yangu katika kunipatia huduma.	❑ Hapana kamwe<br>❑Mara chache<br>❑ Mara nyingi<br>❑ Daima	❑<br>❑<br>❑<br>❑	❑<br>❑<br>❑<br>❑
25.	Ninajisikia huru kuthibitisha ushauri wowote ninaopokea kutoka kwa mhudumu mwingine kwa mhudumu wangu wa afya.	❑ Hapana kamwe<br>❑Mara chache<br>❑ Mara nyingi<br>❑ Daima	❑<br>❑<br>❑<br>❑	❑<br>❑<br>❑<br>❑
26.	Mhudumu wangu wa afya ananielezea kila kitu ninachohitaji kufahamu kuhusu faili langu la matibabu au ananipatia rufaa kwenda kwa daktari.	❑ Hapana kamwe<br>❑Mara chache<br>❑ Mara nyingi<br>❑ Daima	❑<br>❑<br>❑<br>❑	❑<br>❑<br>❑<br>❑
27.	Mhudumu wangu wa afya ananihakikishia usiri kuhusu hali yangu ya VVU.	❑ Hapana kamwe<br>❑Mara chache<br>❑ Mara nyingi<br>❑ Daima	❑<br>❑<br>❑<br>❑	❑<br>❑<br>❑<br>❑
28.	Ninaogopa kumuelezea mhudumu wangu wa afya kuhusu jambo linalotokea kwenye maisha yangu kwa sababu ninahisi atawaambia watu wengine kuhusu jambo hilo.	❑ Hapana kamwe<br>❑Mara chache<br>❑ Mara nyingi<br>❑ Daima	❑<br>❑<br>❑<br>❑	❑<br>❑<br>❑<br>❑

Initial & Date (Research Staff): ____________________ Initial & Date (Data Staff): _________________________

29.	Mhudumu wangu wa afya amepangilia sehemu ya kukutania kwa namna ambayo hakuna mtu mwingine anaweza kusikia nikiwa ninazungumza naye jambo la siri.	❑ Hapana kamwe<br>❑ Mara chache<br>❑ Mara nyingi<br>❑ Daima	❑<br>❑<br>❑<br>❑	❑<br>❑<br>❑<br>❑
**Sehemu B: Matokeo ya Huduma (vipimo vya ubora vya IOM)**				
**Dadisi uzoefu wa wagonjwa katika ziara 3 zilizopita katika kliniki ya huduma na tiba/ vikao vya klabu ya wanaotumia dawa za kupunguza makali ya VVU**				
		**Ziara ya 1**	**Ziara ya 2**	**Ziara ya 3**
30.	**Upatikanaji wa huduma:**<br>Ni rahisi kwangu kuhudhuria kliniki ya huduma na tiba/ kikao cha klabu ya wanaotumia dawa za kupunguza makali ya VVU.	❑ Ndiyo<br>❑ Hapana	❑ Ndiyo<br>❑ Hapana	❑ Ndiyo<br>❑ Hapana
31.	**Mgonjwa kukubalika /kumjali mgonjwa:**<br>Ninapenda jinsi ambavyo ninahudumiwa na wafanyakazi ninapohudhuria huduma za afya.	❑ Ndiyo<br>❑ Hapana	❑ Ndiyo<br>❑ Hapana	❑ Ndiyo<br>❑ Hapana
32.	Ninaridhishwa na namna ambayo mhudumu wangu wa afya hupangilia kliniki ya huduma na tiba/vikao vya klabu ya wanaotumia dawa za kupunguza makali ya virusi.	❑ Ndiyo<br>❑ Hapana	❑ Ndiyo<br>❑ Hapana	❑ Ndiyo<br>❑ Hapana
33.	**Ufanisi:**<br>Mhudumu wangu wa afya hunishawishi nitumie tiba zingine mbadala m.f. tiba za jadi ambazo inanibidi nizilipie.	❑ Ndiyo<br>❑ Hapana	❑ Ndiyo<br>❑ Hapana	❑ Ndiyo<br>❑ Hapana
34.	**Utendaji bora:**<br>Tunatumia muda mwingi kwenye kliniki ya huduma na tiba/vikao vya klabu.	❑ Ndiyo<br>❑ Hapana	❑ Ndiyo<br>❑ Hapana	❑ Ndiyo<br>❑ Hapana
35.	Ninaona kutumia muda wangu kuwa kwenye kliniki ya huduma na tiba/ kikao cha klabu ni kupoteza muda.	❑ Ndiyo<br>❑ Hapana	❑ Ndiyo<br>❑ Hapana	❑ Ndiyo<br>❑ Hapana
36.	Wastani wa muda uliotumika kwenye kliniki ya huduma na tiba/ vikao vya klabu 3 vilivyopita.	Cha kwanza	Cha pili	Cha tatu
37.	**Usawa:**<br>Ninahisi kwamba sihudumiwi vizuri kama wagonjwa wengine ninapokuwa kwenye kliniki ya huduma na tiba/vikao vya klabu ya wanaotumia dawa za kupunguza makali ya virusi.	❑ Ndiyo<br>❑ Hapana	❑ Ndiyo<br>❑ Hapana	❑ Ndiyo<br>❑ Hapana
38.	**Usalama wa mteja:**<br>Ninafahamu aina ya dawa ya kupunguza makali ya virusi ambayo ninaitumia.	❑ Ndiyo<br>❑ Hapana	❑ Ndiyo<br>❑ Hapana	❑ Ndiyo<br>❑ Hapana
39.	Kama jibu ni ndiyo kwenye swali 38, ni aina gani ya dawa? ***(m.f. TDF/FTC/EFV)***<br>*(Muache mgonjwa aelezee namna dawa ilivyo na dozi yake)*			
40.	Ninapokea dawa sahihi za kupunguza makali ya virusi kila ninapokuja.	❑ Ndiyo<br>❑ Hapana	❑ Ndiyo<br>❑ Hapana	❑ Ndiyo<br>❑ Hapana
41.	Nimeshawahi kupewa dawa za kupunguza makali ya VVU ambazo si sahihi.	[ ] Hajawahi	[ ] Mara moja	[ ] >1
42.	Nimewahi kupewa mbadala wa dawa zangu za kupunguza makali ya virusi ninazozitumia.	❑ Ndiyo<br>❑ Hapana		

Initial & Date (Research Staff): ____________________ Initial & Date (Data Staff): _________________________

43.	Kama ni ndiyo kwa swali 42, kwa nini? ***(m.f. dawa zilikwisha, nilipata athari kutokana na dawa nilizokuwa natumia, ujauzito, kifua kikuu n.k.)***			
44.	Ninaweza kuzungumzia wasiwasi nilionao kuhusiana na matibabu yangu kwa uhuru	❑ Ndiyo<br>❑ Hapana	❑ Ndiyo<br>❑ Hapana	❑ Ndiyo<br>❑ Hapana
45.	Mhudumu wangu wa afya huzungumzia kuhusu mabadiliko yoyote katika dawa zangu kwa namna ambayo hainipi hofu	❑ Ndiyo<br>❑ Hapana	❑ Ndiyo<br>❑ Hapana	❑ Ndiyo<br>❑ Hapana
**Sehemu C: Matokeo ya Huduma kutoka kwenye kumbukumbu za wagonjwa kwenye CTC2: (Katika ziara /vikao vya klabu 3 vilivyopita)**		**Ziara ya 1**	**Ziara ya 2**	**Ziara ya 3**
46.	Tarehe ya ziara imeandikwa	[ ]Ndiyo<br>[ ]Hapana	[ ]Ndiyo<br>[ ]Hapana	[ ]Ndiyo<br>[ ]Hapana
47.	Aina ya ziara imeandikwa<br>*(Ziara iliyopangwa – **SV**; Ziara ambayo haikupangwa – **UV**; Kuchukua dawa – **DPU**; Kuongeza dawa kwa ajili ya wagonjwa wenye hali nzuri – **RV**)*	[ ]SV<br>[ ]UV<br>[ ]DPU<br>[ ]RV	[ ]SV<br>[ ]UV<br>[ ]DPU<br>[ ]RV	[ ]SV<br>[ ]UV<br>[ ]DPU<br>[ ]RV
48.	Uzito wa mgonjwa umeandikwa *(andika uzito uliopimwa)*			
49.	Kuna vipimo vyovyote vya uzito visivyo vya kawaida?	[ ]Ndiyo<br>[ ]Hapana	[ ]Ndiyo<br>[ ]Hapana	[ ]Ndiyo<br>[ ]Hapana
50.	Magonjwa nyemelezi yameandikwa	[ ]Ndiyo<br>[ ]Hapana	[ ]Ndiyo<br>[ ]Hapana	[ ]Ndiyo<br>[ ]Hapana
51.	Kuna magonjwa yoyote nyemelezi? *(andika magonjwa nyemelezi yaliyoorodheshwa)*			
52.	Uwezo wa mtu kufanya kazi zake za kila siku umeandikwa	[ ]Ndiyo<br>[ ]Hapana	[ ]Ndiyo<br>[ ]Hapana	[ ]Ndiyo<br>[ ]Hapana
53.	Hali ya ujauzito imewekwa kwenye kumbukumbu (*kwa wagonjwa wa kike*)	[ ]Ndiyo<br>[ ]Hapana	[ ]Ndiyo<br>[ ]Hapana	[ ]Ndiyo<br>[ ]Hapana
54.	Je ni mjamzito? NA – Haihusiani	[ ]Ndiyo<br>[ ]Hapana<br>[ ] Haihusiani	[ ]Ndiyo<br>[ ]Hapana<br>[ ] Haihusiani	[ ]Ndiyo<br>[ ]Hapana<br>[ ] Haihusiani
55.	Uchunguzi wa kifua kikuu umeandikwa	[ ]Ndiyo<br>[ ]Hapana	[ ]Ndiyo<br>[ ]Hapana	[ ]Ndiyo<br>[ ]Hapana
56.	Mgonjwa anadhaniwa kuwa ana kifua kikuu?	[ ]Ndiyo<br>[ ]Hapana	[ ]Ndiyo<br>[ ]Hapana	[ ]Ndiyo<br>[ ]Hapana
57.	Kinga za kuzuia malaria (IPT) zimetolewa na kumbukumbu za kuzitoa kwa mgonjwa zimewekwa	[ ]Ndiyo<br>[ ]Hapana<br>[ ] Haihusiani	[ ]Ndiyo<br>[ ]Hapana<br>[ ] Haihusiani	[ ]Ndiyo<br>[ ]Hapana<br>[ ]Haihusiani
58.	Dawa za kupunguza makali ya virusi zimetolewa na kumbukumbu za kuzitoa kwa mgonjwa zimewekwa	[ ]Ndiyo<br>[ ]Hapana	[ ]Ndiyo<br>[ ]Hapana	[ ]Ndiyo<br>[ ]Hapana
59.	Uzingatiaji wa dawa wa mgonjwa umechunguzwa na kuwekwa kwenye kumbukumbu	[ ]Ndiyo<br>[ ]Hapana	[ ]Ndiyo<br>[ ]Hapana	[ ]Ndiyo<br>[ ]Hapana
60.	Cotrimoxazole imetolewa na kumbukumbu za kuitoa kwa mgonjwa zimewekwa	[ ]Ndiyo<br>[ ]Hapana	[ ]Ndiyo<br>[ ]Hapana	[ ]Ndiyo<br>[ ]Hapana
61.	Hali ya lishe ya mgonjwa imechunguzwa na kumbukumbu zimewekwa	[ ]Ndiyo<br>[ ]Hapana	[ ]Ndiyo<br>[ ]Hapana	[ ]Ndiyo<br>[ ]Hapana
62.	Rufaa za mgonjwa zimewekwa kwenye kumbukumbu ***(Mstari wa rufaa aliyopewa umejazwa)***	[ ]Ndiyo<br>[ ]Hapana	[ ]Ndiyo<br>[ ]Hapana	[ ]Ndiyo<br>[ ]Hapana
63.	Amepewa rufaa kwenda wapi?	[ ] PMTCT	[ ] PMTCT	[ ] PMTCT

	PMTCT *(Kwa mjamzito);* ***Kikundi cha kusaidiana/klabu*** *(msaada wa kundirika);* ***Kliniki ya huduma na tiba*** *(magonjwa nyemelezi/kuumwa);* ***Msaada wa Lishe*** *(kupungua uzito haraka);* ***Kifua kikuu*** *(kwa anayedhaniwa kuwa ana kifua kikuu)*	[ ] Klabu ya ARV<br>[ ] Kliniki ya huduma na tiba<br>[ ] Lishe<br>[ ] Kifua kikuu	[ ] Klabu ya ARV<br>[ ] Kliniki ya huduma na tiba<br>[ ] Lishe<br>[ ] Kifua kikuu	[ ] Klabu ya ARV<br>[ ] Kliniki ya huduma natiba<br>[ ] Lishe<br>[ ] Kifua kikuu
64.	Ufuatiliaji wa mgonjwa umeandikwa	[ ]Ndiyo<br>[ ]Hapana	[ ]Ndiyo<br>[ ]Hapana	[ ]Ndiyo<br>[ ]Hapana
65.	Taarifa zote za ziara ya mgonjwa zimeandikwa *(maeneo yote yanayotakiwa kujazwa kwenye daftari la kumbukumbu yamejazwa)*	[ ]Ndiyo<br>[ ]Hapana	[ ]Ndiyo<br>[ ]Hapana	[ ]Ndiyo<br>[ ]Hapana
66.	Kiwango cha CD4 kipindi ambacho mgonjwa alianzishiwa dawa za kupunguza makali ya virusi *(andika kiwango hicho)*			
67.	Kiwango cha CD4 katika kipindi cha miezi 6 iliyopita *(andika kiwango hicho)*			
68.	Kipimo cha wingi wa virusi katika kipindi cha miezi 6 iliyopita	[ ]Ndiyo<br>[ ]Hapana	[ ]Ndiyo<br>[ ]Hapana	[ ]Ndiyo<br>[ ]Hapana
69.	Majibu ya kipimo cha wingi wa virusi (*andika majibu hayo)*			

Yafuatayo ni maswali kuhusu huduma unazopata kutoka kwa mhudumu wako wa afya kwa ujumla.

224. Je, huduma unayopata kutoka kwa mhudumu wako wa afya ni:
- □ Ya kuridhisha
- □ Hairidhishi
- □ Sio nzuri wala mbaya

225. Weka alama ya (X) hapa chini kuonyesha ni kiasi gani kwa ujumla unaridhishwa na huduma uliyopokea kutoka kwa mhudumu wako wa afya katika kipindi cha miezi 6 iliyopita, kuhusiana na maambukizi yako ya VVU
- □ Nimeridhishwa
- □ Nimeridhishwa sana
- □ Sijaridhishwa
- □ Sijaridhishwa kabisa

Initial & Date (Research Staff): ____________________ Initial & Date (Data Staff): _________________________
