# Supplementary material for: Quality of care in a differentiated HIV service delivery intervention in Tanzania: A mixed-methods study
Source: PLoS One. 2022 Mar 15;17(3):e0265307. doi: 10.1371/journal.pone.0265307 (PMC8923447; doi:10.1371/journal.pone.0265307)
Supplement: Attachment — Submitted filename: 2022.02.25_Response to Reviewers.docx [file pone.0265307.s004.docx]

**Response to Reviewers**

**Academic editor**

Dear Dr. Okere,

Thank you for submitting your manuscript to PLOS ONE. After careful consideration, we feel that it has merit but does not fully meet PLOS ONE’s publication criteria as it currently stands. Therefore, we invite you to submit a revised version of the manuscript that addresses the points raised during the review process.

One of the reviewers has made some valuable point regarding the format of the manuscript and myself as the Academic Editor appreciate their perspective. In this case, all major and minor concerns have been indeed addressed by you, in your rebuttal and the revised manuscript in a satisfactory way. However, I would ask that you make one more effort to increase the readability of your manuscript and improve its formatting to account for the mixed methods approach you have -rightly- undertaken.  I would appreciate if you consult and cite any methodological source(s) in your references. I have personally found particularly helpful most of the methodological papers and manuals by John W Creswell and his team, but there are several others.

Thank you for another opportunity to revise our paper further and make it more readable.

1. We have reformatted the entire results section in a bid to improve flow and accentuate the information gleaned from our mixed methods approach. After trying out various options, we decided to maintain the structure of the Donabedian framework – structure, process, and outcome. In each part, first, we present the quantitative findings from clients, followed by their qualitative findings. Then we present the quantitative findings from HCW, followed by their qualitative findings. We repeat the same format for all three parts.

We also renamed the Figures and Tables in an attempt to clarify to which part they belong and tried grouping them as much as possible to improve readability. While revising we came across some minor textual things which we took the liberty of changing; please see the tracked changes manuscript.

2. We have indeed perused the remarkable work done by John W Creswell and a couple of others and based on recommendations for highlighting the main findings for mixed methods research in joint display tables, have created such a table that we have included in our paper, see table 3d. The table summarizes the main findings for the quantitative and qualitative parts. We have included the explanation of the ordering of the results and the inclusion of the joint display table in the methods section, see lines 107-111. We have also included the appropriate citations of the methodology employed.

**Reviewer #1:**

The authors have adequately addressed all minor comments and clarifying questions.
However, while mixed methods approaches bear strengths in triangulating information - careful thought and tact should be given to how the results are presented. We understand that the authors did not want to tone down the findings. However, at the same time - the value of the work seems to be lost in how the results section is currently organised. I would recommend that authors reconsider formatting the results section as suggested by both reviewers. The current revisions made do not adequately address this concern.

Thank you for your feedback. We have taken your recommendations and reformatted the entire results section as suggested. We hope that we have adequately addressed your concern by our revision.
